# Supplementary material for: Morphological and Quantitative Evidence for Altered Mesenchymal Stem Cell Remodeling of Collagen in an Oxidative Environment—Peculiar Effect of Epigallocatechin-3-Gallate
Source: Polymers (Basel). 2022 Sep 22;14(19):3957. doi: 10.3390/polym14193957 (PMC9571090; doi:10.3390/polym14193957)
Supplement: Supplementary file 1 [file polymers-14-03957-s001.zip › polymers-1888266-supplementary.pdf]

Supplementary material for the manuscript entitled

"Morphological and quantitative evidence for altered mesenchymal stem cell remodeling of collagen in an oxidative environment – peculiar effect of epigallocatechin-3-gallate" to be considered for publication in "Polymers", Special issue "Polymer Materials in Biomedical Application II".

Table S1. Total anisotropy index (AI) of FITC labelled collagen samples, consisting of native (MTC), oxidized (MTC-OXI) and MTC-OXI pretreated with EGCG (MTC OXI/EGCG) measured for bare samples (- cells) and for samples (+ cells). The change of anisotropy index by the cells is presented as Δ AI. More detailed measurements of the same parameters performed in the selected regions of interest (ROI): Outside the cells, the Cellular region, and the Central part which includes the nuclear/perinuclear cell region.

Collagen Sample	Total anisotropy (- cells)	Total anisotropy (+ cells)	Change in the anisotropy (ΔAI)	Region of interest (ROI)	Anisotropy of the region
MTC	0.069	0.212	0.143	Outside Cell	0.009
				Cell region	0.383
				Central part	0.119
MTC OXI	0.028	0.116	0.088	Outside Cell	0.020
				Cell region	0.448
				Central part	0.553
MTC OXI/EGCG	0.053	0.083	0.031	Outside Cell	0.060
				Cell region	0.374
				Central part	0.381

Table S2. Total area in pixels, Type of regions of interest (ROI), Mean of fluorescence intensity (MFI) in pixels and Standard deviation (SD) of FITC collagen studied in different ROI: Outside the cells, the Cellular region, and the Central part. The native (MTC), oxidized (MTC-OXI), and EGCG pretreated (MTC-OXI/EGCG) collagen samples were measured and compared. The ratio Inside/Outside the cells of MFI for Cellular region and the Central part versus Outside the cells was calculated for the same collagen samples.

Sample	Total area (pixels)	Type ROI	MFI (pixels)	SD	Ratio Inside/Outside the cells
MTC	1904820	Outside Cell	4.88	1.85	
		Cellular region	5.41	2.56	1.11
		Central part	5.73	1.94	1.17
MTC-OXI	1878228	Outside Cell	3.03	0.80	
		Cellular region	3.58	1.47	1.18
		Central part	4.01	1.48	1.32
MTC-OXI/EGCG	1893672	Outside Cell	4.48	0.96	
		Cellular region	5.62	1.44	1.26
		Central part	7.02	1.14	1.57
